# Case Report: Uremic stomatitis revealing undiagnosed kidney failure in an adolescent with PAX2-related nephropathy

**DOI:** 10.3389/fped.2026.1892668

**Published:** 2026-10-29

**Authors:** Domenico De Falco, Doriana Pedone, Laura Roselli, Alessandra Caggiula, Nicol Macripò, Pasquale Sportelli, Giovanni FM Strippoli, Massimo Petruzzi

**Affiliations:** 1 Interdisciplinary Department of Medicine, University of Bari “Aldo Moro”, Bari, Italy; 2 Department of Precision and Regenerative Medicine and Ionian Area (DIMEPRE-J), University of Bari “Aldo Moro”, Bari, Italy

**Keywords:** chronik kidney disease, oral medicine, renal failure, tongue, uremic stomatitis

## Abstract

**Background:**

Uremic stomatitis is a rare oral mucosal disorder associated with kidney failure in adults. It is exceptionally uncommon in children. Its clinical presentation may mimic more common conditions, such as oral candidiasis or traumatic keratosis, potentially delaying recognition of the underlying systemic disease. To our knowledge, this appears to be the first reported pediatric case of uremic stomatitis associated with PAX2-related nephropathy.

**Case presentation:**

A 15-year-old female patient was referred for persistent multifocal white lesions of the oral mucosa. Intraoral examination revealed pseudomembranous and hyperkeratotic whitish plaques involving the right buccal mucosa, labial commissure, floor of the mouth, and the ventral and lateral surfaces of the tongue, with partially removable areas associated with underlying erythema and superficial erosions. The lesions showed only partial improvement following topical antifungal and antiseptic therapy. Their persistence, diffuse distribution, and the presence of marked mucosal pallor prompted further laboratory investigations. Blood tests revealed severe anemia (hemoglobin 5.4 g/dL), elevated serum urea (106 mg/dL), serum creatinine of approximately 13 mg/dL, severely reduced estimated glomerular filtration rate (approximately 3.7 mL/min/1.73 m^2^), and hypocalcemia. The patient was urgently referred to Pediatric Nephrology and diagnosed with kidney failure, subsequently attributed to PAX2-related nephropathy. Renal replacement therapy with hemodialysis was initiated, followed by progressive regression of the oral lesions. The patient later underwent kidney transplantation with favorable early graft recovery.

**Conclusion:**

This case highlights uremic stomatitis as a rare but clinically significant oral manifestation of kidney failure also in children and emphasizes the importance of considering systemic disorders in the differential diagnosis of persistent multifocal white oral lesions. In particular, lesions associated with mucosal pallor and poor response to conventional therapy should prompt laboratory investigations, including renal function assessment. This report further underscores the important role of oral medicine specialists in the early recognition of occult systemic disease and timely referral for multidisciplinary management.

## Introduction

Chronic kidney disease (CKD) in children is an uncommon but clinically significant condition associated with substantial long-term morbidity and multisystem complications. In contrast to adult CKD, which is predominantly related to acquired disorders such as diabetes and hypertension, pediatric CKD is more frequently associated with congenital and hereditary nephropathies, including congenital anomalies of the kidney and urinary tract (CAKUT) and genetic renal disorders (1–3). Oral manifestations may occur during the course of CKD, particularly in advanced stages of disease, and may reflect the combined effects of uremia, anemia, metabolic disturbances, immune dysfunction, altered salivary composition, and kidney replacement therapy (2, 4, 5). Reported oral findings in pediatric patients with CKD include enamel defects, delayed tooth eruption, xerostomia, dysgeusia, gingival and periodontal alterations, mucosal pallor, opportunistic infections, and oral mucosal lesions (2). Uremic stomatitis is a rare oral mucosal disorder classically associated with advanced or untreated kidney failure. Although first described in the nineteenth century, only a limited number of cases have been reported in the literature, most involving adults (6, 7). A recent literature review identified 52 published cases overall, of which only three involved children or adolescents (5.8%), underscoring the exceptional rarity of pediatric presentations (7). The pathogenesis of uremic stomatitis remains incompletely understood. Salivary urea hydrolysis into ammonia is considered the main mechanism of mucosal injury (6–8), while xerostomia, anemia, impaired tissue repair, reduced mucosal resistance, and secondary infections may also contribute (7, 8). Clinically, uremic stomatitis may present as ulcerative, hemorrhagic, pseudomembranous, or hyperkeratotic lesions involving the tongue, buccal mucosa, floor of the mouth, or labial mucosa (4, 6, 7). However, its diagnosis may be particularly challenging because the clinical appearance frequently overlaps with more common oral disorders, including oral candidiasis, traumatic keratosis, oral hairy leukoplakia, chemical mucositis, and immune-mediated mucosal diseases (7, 9). This diagnostic overlap may delay recognition of underlying renal dysfunction, especially in pediatric patients without a previous history of kidney disease. Despite increasing awareness of oral manifestations associated with CKD, pediatric uremic stomatitis remains poorly characterized in the literature, with only isolated case reports currently available. Furthermore, diagnostic pathways linking oral findings to previously unrecognized hereditary kidney disease have rarely been discussed. We report a rare pediatric case of uremic stomatitis that represented the initial clinical manifestation of previously undiagnosed kidney failure secondary to PAX2-related nephropathy, highlighting the importance of timely multidisciplinary referral.

### Case description

A 15-year-old female patient was referred to the Oral Medicine Department of the University of Bari, Italy, for evaluation of persistent multifocal white lesions involving the oral mucosa. The patient reported habitual biting of the right buccal mucosa and tongue. At the time of the initial evaluation, no relevant drug allergies, recent medication intake, or previously known systemic diseases were reported. The patient did not report halitosis, dysgeusia, or weight loss. No neurological manifestations attributable to uremia, such as confusion, altered mental status, or seizures, were reported or clinically observed, and no clinical signs suggestive of hepatic involvement were documented. Intraoral examination revealed multifocal whitish plaques involving the right buccal mucosa, labial commissure, floor of the mouth, and the ventral and lateral surfaces of the tongue (Figure 1). The lesions showed combined pseudomembranous and hyperkeratotic features, with partially detachable areas revealing underlying erythematous and superficially eroded mucosa. No significant cervical lymphadenopathy was detected. Based on the initial clinical presentation, oral candidiasis and traumatic keratosis were considered in the differential diagnosis. Empirical topical therapy with miconazole oral gel (20 mg/g, applied three to four times daily) and 0.12% chlorhexidine mouthwash after oral hygiene was prescribed for six days, until the first follow-up visit. At follow-up evaluation, the lesions showed only partial improvement after antifungal therapy. Persistent multifocal whitish plaques remained evident, particularly involving the tongue and oral mucosa, and their clinical appearance was considered atypical for uncomplicated oral candidiasis or isolated traumatic keratosis. In particular, the diffuse distribution of the lesions, partial removability, and incomplete therapeutic response raised suspicion of an underlying systemic disorder. In addition, marked mucosal pallor was observed during examination. Consequently, laboratory investigations, including complete blood count and renal function tests, were requested. Given the marked mucosal pallor and suspicion of an underlying systemic disorder, blood tests were prioritized over incisional biopsy, as they represented a more rapid and minimally invasive investigation, with results available the following day. Biopsy was therefore deferred at that stage. Laboratory investigations revealed severe anemia (hemoglobin 5.4 g/dL), markedly elevated serum urea (106 mg/dL), serum creatinine of approximately 13 mg/dL, and a severely reduced estimated glomerular filtration rate of 3.78 mL/min/1.73 m^2^. Additional abnormalities included reduced serum iron (30 µg/dL), severe hypocalcemia (total serum calcium 5.4 mg/dL), elevated parathyroid hormone levels (406 pg/mL), reduced 25-hydroxyvitamin D levels (13 ng/mL), and markedly elevated *β*2-microglobulin (22.8 mg/L). Urinalysis showed marked proteinuria (approximately 300 mg/dL), a urinary pH of 7.5, a specific gravity of 1.007, and microscopic hematuria.

**Figure 1:**
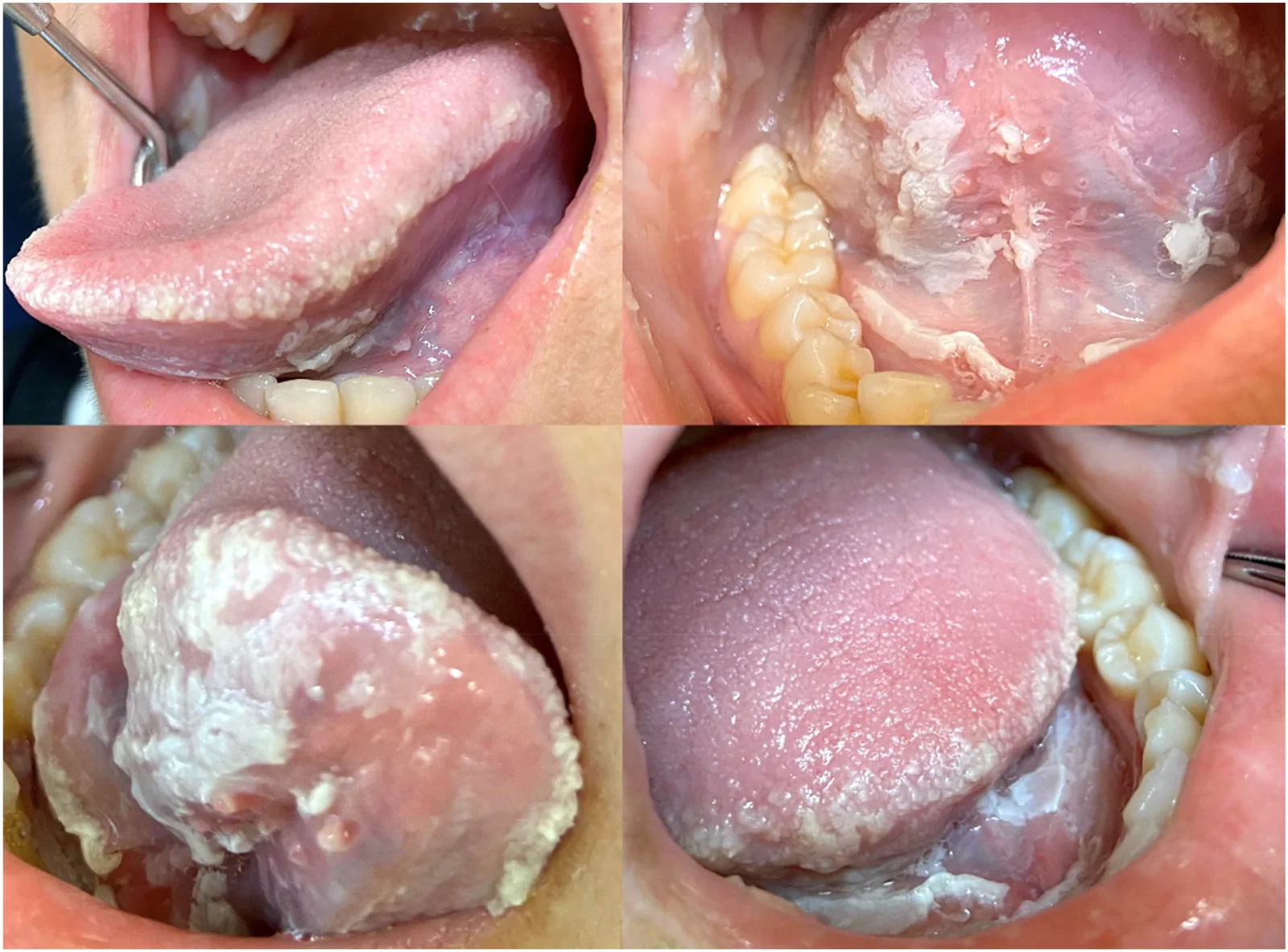
Multifocal hyperkeratotic and pseudomembranous whitish plaques involving the ventrolateral tongue, floor of the mouth, and buccal mucosa. Some lesions were partially removable, revealing underlying erythematous and superficially eroded mucosa.

These findings prompted urgent referral to the Pediatric Nephrology Unit, where the patient was admitted and diagnosed with kidney failure requiring renal replacement therapy. Following the abnormal laboratory findings, a more detailed family history was obtained, revealing that the patient's twin sister had CKD and had previously undergone kidney transplantation. During kidney specialist evaluation, the patient was initially classified as having kidney failure secondary to an undefined familial nephropathy.

The available nephrology documentation reported the identification of a PAX2 mutation and indicated that the patient's twin sister carried the same mutation. However, the sequencing method used was not specified in the nephrology consultation report.

Hemodialysis was initiated through a central venous catheter. Progressive regression of the oral lesions was subsequently observed following correction of the uremic state, further supporting the diagnosis of uremic stomatitis. The patient subsequently underwent multidisciplinary evaluation for kidney transplantation. Imaging demonstrated small hyperechogenic kidneys consistent with advanced CKD, while no odontogenic infectious foci were identified, although professional dental debridement was recommended. After discharge, thrice-weekly hemodialysis was continued until January 2026, when the patient underwent deceased-donor kidney transplantation with favorable early graft recovery and progressive restoration of renal function.

Induction immunosuppression consisted of basiliximab and intravenous methylprednisolone, followed by maintenance therapy with tacrolimus, mycophenolate mofetil, and corticosteroids.

The dialysis catheter and ureteral stent were subsequently removed during follow-up. The temporal association between severe uremia, initiation of renal replacement therapy, and progressive regression of the oral lesions strongly supported the diagnosis of uremic stomatitis (Table 1).

**Table 1:** Timeline of the patient's clinical course.

Time point	Oral findings	Key systemic findings	Management/outcome
November 2024	Multifocal pseudomembranous-hyperkeratotic whitish plaques involving the tongue, buccal mucosa, floor of mouth, and labial commissure	No known kidney disease; no major systemic symptoms	Initial suspicion of oral candidiasis/traumatic keratosis; topical miconazole+chlorhexidine prescribed
November 2024 (follow-up)	Partial improvement after antifungal therapy; persistent diffuse white plaques and mucosal pallor	Suspicion of underlying systemic disease	Complete blood count and renal function tests requested
December 2024/January 2025	Persistent multifocal oral lesions	Severe anemia (Hb 5.4 g/dL), serum urea 106 mg/dL, creatinine 13 mg/dL, eGFR 3.7 mL/min/1.73 m^2^, hypocalcemia	Urgent referral to Pediatric Nephrology Unit
January 2025	Regression of oral lesions after initiation of renal replacement therapy	End-stage kidney disease; suspected familial nephropathy	Hemodialysis initiated via central venous catheter
January–February 2025	Continued oral improvement	Small hyperechogenic kidneys consistent with advanced CKD; no odontogenic infectious foci	Multidisciplinary transplant assessment; professional dental debridement recommended
2025	Progressive resolution of oral lesions	PAX2-related nephropathy confirmed by genetic/clinical investigations	Continuation of thrice-weekly hemodialysis and transplant follow-up
January 2026	No recurrence of oral lesions	Favorable early graft recovery after kidney transplantation	Deceased-donor kidney transplantation; immunosuppressive therapy initiated
March 2026	Stable oral condition	Progressive restoration of renal function	Removal of dialysis catheter and ureteral stent

CKD, chronic kidney disease; eGFR, estimated glomerular filtration rate; Hb, hemoglobin.

## Discussion

This case illustrates how persistent multifocal white oral lesions may represent the first clinically evident manifestation of previously undiagnosed kidney failure in children. Although uremic stomatitis is a rare entity, particularly in children and adolescents, failure to recognize this condition may delay kidney specialist evaluation and the initiation of potentially life-saving renal replacement therapy (10). In the present patient, oral findings preceded the diagnosis of advanced kidney disease and prompted systemic investigations that ultimately revealed severe uremia and hereditary nephropathy. The pathogenesis of uremic stomatitis remains incompletely understood. The most widely accepted mechanism involves increased salivary urea concentrations that undergo bacterial urease-mediated hydrolysis into ammonia, resulting in chemical irritation and epithelial injury of the oral mucosa (6–8). Additional contributing factors may include xerostomia, impaired mucosal repair, secondary infections, anemia-associated tissue hypoxia, immune dysfunction, and reduced mucosal resistance associated with advanced uremia (7, 8). In the present case, the severe azotemia, profound anemia, and marked metabolic impairment may all have contributed to the development of the pseudomembranous-hyperkeratotic oral lesions. In particular, anemia likely contributed to the marked mucosal pallor and may have reduced mucosal resistance, potentially favoring secondary Candida overgrowth. However, the multifocal pseudomembranous-hyperkeratotic presentation was not considered fully attributable to anemia alone. In advanced CKD, anemia is typically multifactorial and may reflect relative erythropoietin deficiency, impaired red blood cell survival, iron deficiency, and inflammation (11, 12).

Clinically, the diagnosis of uremic stomatitis may be particularly challenging because its presentation overlaps with more common oral disorders, including oral candidiasis, traumatic keratosis, immune-mediated mucosal diseases, oral hairy leukoplakia, and chemical or contact mucositis (7, 9). In this patient, the history of habitual cheek and tongue biting initially supported consideration of traumatic keratosis, while the partially removable whitish plaques suggested oral candidiasis. However, the diffuse multifocal distribution of the lesions, incomplete response to antifungal therapy, marked mucosal pallor, and persistence of lesions after initial treatment were considered atypical for uncomplicated local oral disease and raised suspicion of an underlying systemic disorder. This diagnostic overlap emphasizes the importance of maintaining a broad differential diagnosis when evaluating persistent white oral lesions in pediatric patients (Table 2).

**Table 2:** Differential diagnosis of persistent multifocal white oral lesions in the present case.

Differential diagnosis	Findings supporting the diagnosis	Findings arguing against the diagnosis
Oral candidiasis	Presence of pseudomembranous whitish plaques with partially removable areas and underlying erythema.	Only partial response to antifungal therapy; diffuse multifocal distribution and persistence of lesions were atypical for uncomplicated candidiasis.
Traumatic keratosis	History of habitual biting of the tongue and right buccal mucosa; hyperkeratotic appearance of some lesions.	Lesions involved multiple oral sites, including the floor of the mouth and ventrolateral tongue, which was disproportionate to isolated mechanical trauma.
Oral hairy leukoplakia	Whitish plaques involving the lateral tongue.	Absence of known immunosuppression or Epstein–Barr virus-related disease; partially detachable lesions and associated erosive areas were not typical.
Chemical or contact mucositis	Presence of erythematous and superficially eroded areas.	No history of exposure to caustic agents, irritative substances, or recent changes in oral hygiene products or medications.
Immune-mediated mucosal disease	Multifocal oral involvement and erosive component.	Lack of characteristic reticular, bullous, or ulcerative patterns and absence of extraoral manifestations.
Uremic stomatitis	Multifocal pseudomembranous-hyperkeratotic lesions, marked mucosal pallor, severe renal impairment, anemia, and progressive regression after initiation of hemodialysis.	Exceptional rarity in pediatric patients and absence of previous diagnosis of kidney disease initially reduced clinical suspicion.

EBV, Epstein–Barr virus.

In persistent, atypical, or diagnostically uncertain lesions, an incisional biopsy should also be considered, as histopathological examination may facilitate the early exclusion of alternative mucosal disorders and potentially expedite the identification of an underlying systemic condition (13). In the present case, however, biopsy was deferred because the marked mucosal pallor and the overall clinical presentation raised immediate concern for an underlying systemic disorder, leading to prioritization of laboratory investigations. The subsequent detection of severe kidney failure and the progressive regression of the oral lesions following hemodialysis strongly supported the clinical diagnosis and reduced the need for histopathological confirmation.

A suggested diagnostic approach to persistent multifocal white oral lesions in pediatric patients is summarized in Figure 2. Although uremic stomatitis is uncommon, dentists and oral medicine specialists may play a critical role in the early recognition of occult systemic disease by identifying oral findings that warrant further medical investigation.

**Figure 2:**
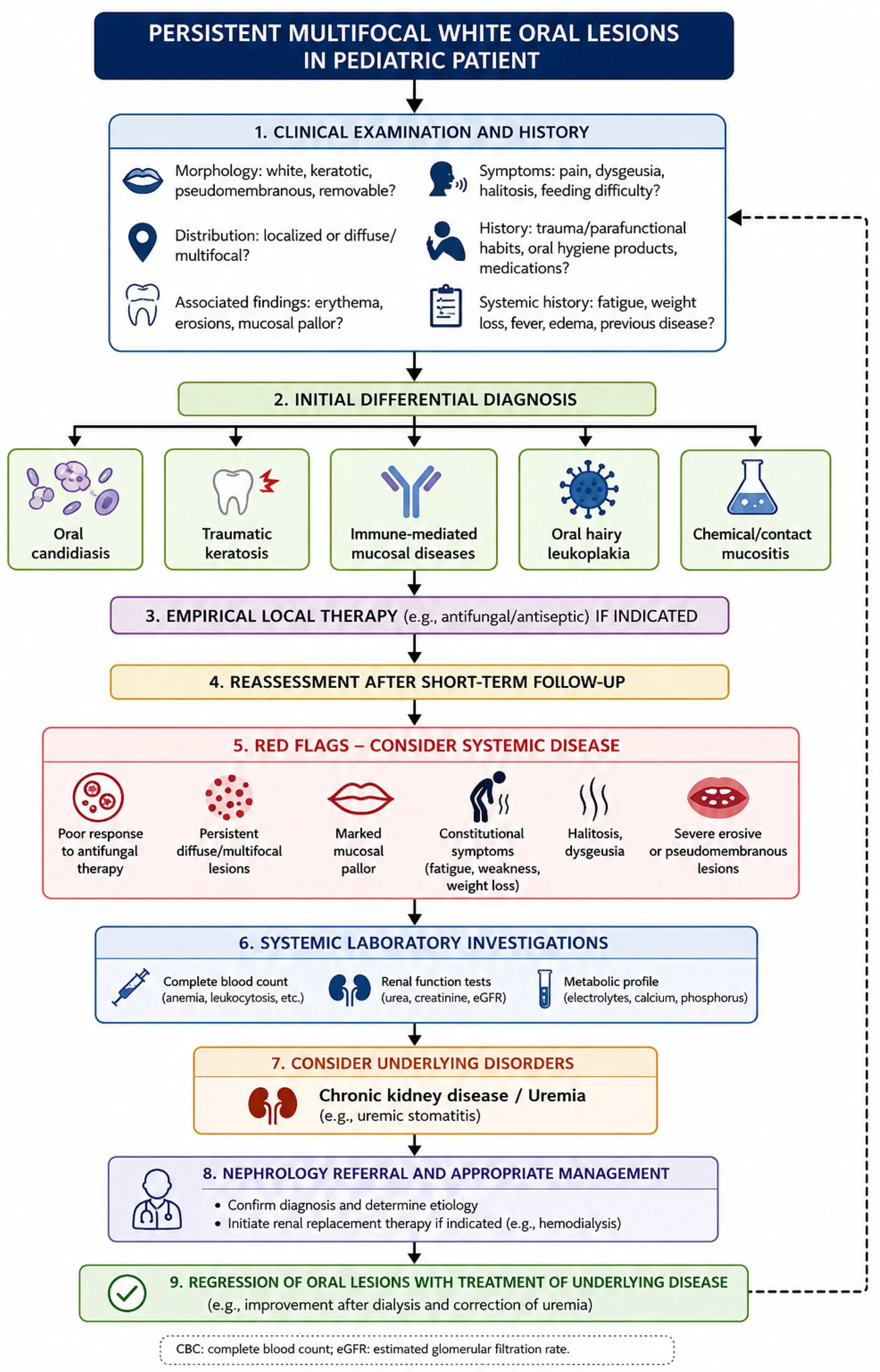
Suggested diagnostic approach to persistent multifocal white oral lesions in pediatric patients. The algorithm outlines a stepwise diagnostic pathway beginning with comprehensive clinical examination and history taking, including assessment of lesion morphology, distribution, associated symptoms, parafunctional habits, and systemic findings. Initial differential diagnoses include oral candidiasis, traumatic keratosis, immune-mediated mucosal diseases, oral hairy leukoplakia, and chemical/contact mucositis. In cases showing poor response to empirical local therapy, persistent diffuse or multifocal lesions, marked mucosal pallor, constitutional symptoms, halitosis/dysgeusia, or severe erosive-pseudomembranous lesions, systemic laboratory investigations including complete blood count, renal function tests, and metabolic profile are recommended. Identification of renal impairment should prompt consideration of chronic kidney disease and uremic stomatitis, with timely nephrology referral and appropriate management. Regression of oral lesions following correction of the uremic state further supports the diagnosis of uremic stomatitis. CBC, complete blood count; eGFR, estimated glomerular filtration rate.

Comparison with previously reported pediatric cases highlights similarities in presentation but differences in renal context and outcome. Ross and Salisbury reported a similar presentation in a 13-year-old boy with white oral plaques and previously undiagnosed renal failure (14). His reported lesions involved the ventral tongue, whereas our patient showed multifocal pseudomembranous–hyperkeratotic lesions involving several oral sites (14). As summarized by de Arruda et al., his systemic findings included anemia, hypertension, elevated BUN (174 mg/dL) and creatinine (16.7 mg/dL), and hypocalcemia (4.3 mg/dL); hemodialysis was followed by partial resolution of the lesions, without a specified regression interval in the review (7). Hasselfeld et al. described a 16-year-old boy whose white tongue was initially treated as presumed candidiasis before investigations revealed previously unrecognized end-stage kidney disease, subsequently attributed to dysfunctional voiding (Hinman syndrome) (10). Laboratory findings included BUN of 180 mg/dL, creatinine of 24.31 mg/dL, hemoglobin of 4.6 g/dL, and calcium of 3.4 mg/dL. Urgent hemodialysis in the pediatric intensive care unit was followed by resolution of uremic stomatitis within four days (10). Similarly, in our patient, persistent lesions after empirical antifungal treatment and marked mucosal pallor prompted systemic investigations, revealing severe anemia (hemoglobin 5.4 g/dL), renal impairment (serum urea 106 mg/dL; creatinine approximately 13 mg/dL), and hypocalcemia (5.4 mg/dL). Subsequent nephrological assessment identified PAX2-related nephropathy. In contrast, the 14-year-old girl reported by Piliang et al. had already diagnosed kidney failure attributed to glomerulopathy and was undergoing hemodialysis at oral assessment (15). Her presentation included painful plaques, pseudomembranes, erosions, purpura, and feeding difficulties, with anemia and elevated urea and creatinine (15). Oral hygiene measures, chlorhexidine, saline, and supportive lip treatment were associated with disappearance of intraoral lesions by the third visit, although residual lip dryness persisted at the fourth visit (15). Our patient showed progressive regression after hemodialysis and subsequently underwent kidney transplantation in January 2026, with favorable early graft recovery and no recurrence of the oral lesions.

The limited number of pediatric reports likely reflects the relative rarity of end-stage kidney disease in children compared with adults (16, 17). In pediatric populations, CKD is more commonly associated with congenital and hereditary disorders, particularly CAKUT-spectrum abnormalities and genetic nephropathies, whereas adult CKD more frequently results from acquired conditions such as diabetes mellitus and hypertension (16, 17). To our knowledge, the present report is among the very few pediatric cases of uremic stomatitis currently described in the literature and appears to be the first associated with PAX2-related nephropathy.

PAX2-related nephropathy is a hereditary disorder characterized by abnormal renal development and progressive CKD. Clinical manifestations may be heterogeneous and include CAKUT-spectrum abnormalities, renal hypoplasia, and familial nephropathy. Although oral manifestations secondary to advanced uremia have been described in CKD, uremic stomatitis associated with PAX2-related disease has not previously been reported. In the present case, oral lesions represented an early clinical clue leading to the diagnosis of previously unrecognized hereditary end-stage kidney disease. In this patient, the temporal association between severe uremia, initiation of hemodialysis, and progressive regression of the oral lesions strongly supported the diagnosis of uremic stomatitis.

Finally, reports from Italy suggest that access to specialist oral medicine and oral pathology services may be constrained by their concentration in a limited number of hospital and university centres, while European survey data indicate heterogeneity in oral medicine training and clinical practice. The implications of these observations for the recognition of rare conditions such as uremic stomatitis remain to be established. This case further emphasizes the importance of interdisciplinary collaboration between dentists, oral medicine specialists, pediatricians, and nephrologists in the evaluation of persistent oral mucosal lesions in children (18, 19).

This case further emphasizes the importance of interdisciplinary collaboration between dentists, oral medicine specialists, pediatricians, and nephrologists in the evaluation of persistent oral mucosal lesions in children.

This case report has several limitations. First, no oral biopsy or mycological investigations, including fungal cytology or culture, were performed. Consequently, concomitant oral candidiasis cannot be excluded, particularly given the six-day course of empirical miconazole treatment. The diagnosis of uremic stomatitis was clinical, supported by the identification of severe kidney failure and subsequent regression of the oral lesions following hemodialysis. Furthermore, post-dialysis clinical photographs were not available to document the regression of the oral lesions. The original genetic testing report was unavailable to the authors; consequently, the precise PAX2 variant and the sequencing method could not be verified. Finally, as this report describes a single patient, its findings may not be generalizable to the broader pediatric population with advanced kidney disease.

## Conclusions

This case highlights uremic stomatitis as a rare but clinically significant oral manifestation of advanced kidney disease in pediatric patients and emphasizes the importance of considering systemic disorders in the differential diagnosis of persistent multifocal white oral lesions. In particular, pseudomembranous or hyperkeratotic plaques associated with mucosal pallor, dysgeusia, halitosis, feeding difficulties, constitutional symptoms, or poor response to empirical antifungal therapy should prompt systemic evaluation, including complete blood count and renal function testing (15).

To our knowledge, this appears to be the first reported pediatric case of uremic stomatitis associated with PAX2-related nephropathy presenting as the initial clinical clue of previously undiagnosed end-stage kidney disease. This report further underscores the important role of dentists and oral medicine specialists in the early recognition of occult systemic disease and timely multidisciplinary referral (7, 18, 19). Early identification of uremic stomatitis may facilitate prompt nephrological assessment and initiation of renal replacement therapy, potentially reducing the risk of severe complications in affected children (10, 15).

## Data Availability

The raw data supporting the conclusions of this article will be made available by the authors, without undue reservation.
